# Earliest known lepisosteoid extends the range of anatomically modern gars to the Late Jurassic

**DOI:** 10.1038/s41598-017-17984-w

**Published:** 2017-12-19

**Authors:** Paulo M. Brito, Jésus Alvarado-Ortega, François J. Meunier

**Affiliations:** 1 Departamento de Zoologia, Instituto de Biologia, Universidade do Estado do Rio de Janeiro, Rua São Francisco Xavier, 524, 20550–900, Rio de Janeiro, Brazil; 2 Departamento de Paleontología, Instituto de Geología, Universidad Nacional Autónoma de México, Ciudad Universitaria, Coyoacán, Distrito Federal, 04510 Mexico; 3 UMR 7208 (CNRS-IRD-MNHN-UPMC), Biologie des Organismes et Ecosystèmes Aquatiques, Département Adaptations du Vivant, Muséum National d’Histoire Naturelle, 43 rue Cuvier, 75231 Paris, France

## Abstract

Lepisosteoids are known for their evolutionary conservatism, and their body plan can be traced at least as far back as the Early Cretaceous, by which point two families had diverged: Lepisosteidae, known since the Late Cretaceous and including all living species and various fossils from all continents, except Antarctica and Australia, and Obaichthyidae, restricted to the Cretaceous of northeastern Brazil and Morocco. Until now, the oldest known lepisosteoids were the obaichthyids, which show general neopterygian features lost or transformed in lepisosteids. Here we describe the earliest known lepisosteoid (*Nhanulepisosteus mexicanus* gen. and sp. nov.) from the Upper Jurassic (Kimmeridgian – about 157 Myr), of the Tlaxiaco Basin, Mexico. The new taxon is based on disarticulated cranial pieces, preserved three-dimensionally, as well as on scales. *Nhanulepisosteus* is recovered as the sister taxon of the rest of the Lepisosteidae. This extends the chronological range of lepisosteoids by about 46 Myr and of the lepisosteids by about 57 Myr, and fills a major morphological gap in current understanding the early diversification of this group.

## Introduction

Actinopterygians, or ray-finned fishes, are the largest group among extant gnathostoms vertebrates. Today actinopterygians are represented by three major clades: Cladistia (bichirs and rope fish), with at least 16 species, Chondrostei (sturgeons and paddle fishes), with about 30 species, and Neopterygii, formed by the Teleostei, with about 30,000 species and the Holostei with eight species: one halecomorph (bowfin) and 7 ginglymodians (gars)^1^. Although neopterygians have a deep evolutionary history, dating back to the Palaeozoic^2–4^, their most important radiation occurred in the early Mesozoic^5,6^ when they began to dominate aquatic ecosystems and when the holosteans were much diverse, presenting a taxonomic richness that exceeded that of teleosts^7^. Among the holosteans, ginglymodians represent a specialized clade, the relationships of which have recently been reassessed^8–10^.

Gars are the only extant ginglymodians and represent a lineage that goes back into the Mesozoic, when they were known by two families: the group of modern gars, the Lepisosteidae, known since the Late Cretaceous, and with known fossils from all continents, except Antarctica and Australia^11^, and the Obaichthyidae, an extinct group until now restricted to the Early Cretaceous of northeastern Brazil and eastern Morocco. Obaichthyids retain a number of plesiomorphic characters including a free maxilla with a well-developed anterior articular process, an interopercle, absence of lacrimomaxillary bones, absence of plicidentine tooth structure, and a layer of dentine in the scales between the lamellar bone and the ganoin layer^11–13^. The previous oldest known fossil gars, the obaichthyid *Dentilolepisosteus laevis* Wenz and Brito^12^, derive from 100 million years old deposits of Brazil (Lower Cretaceous)^14^.

Here, we report the discovery of a new fossil lepisosteid from the Late Jurassic of Mexico, recognized mostly from cranial remains, which extends the record of the family back by an additional ~57 million years. This taxon shares features with fossil and living gars, filling a major morphological gap in understanding the early diversification of this group. The new taxon shows that, already in the Late Jurassic, lepisosteids had developed their distinctive anatomical body plan, most notably in the geometry of the skull.

## Results

**Systematic palaeontology**.

Neopterygii Regan, 1923

Ginglymodii Cope, 1872

Lepisosteiformes Hay, 1929

Lepisosteoidea López-Arbarello, 2012

Lepisosteidae Cuvier, 1825

**Figure 1:**
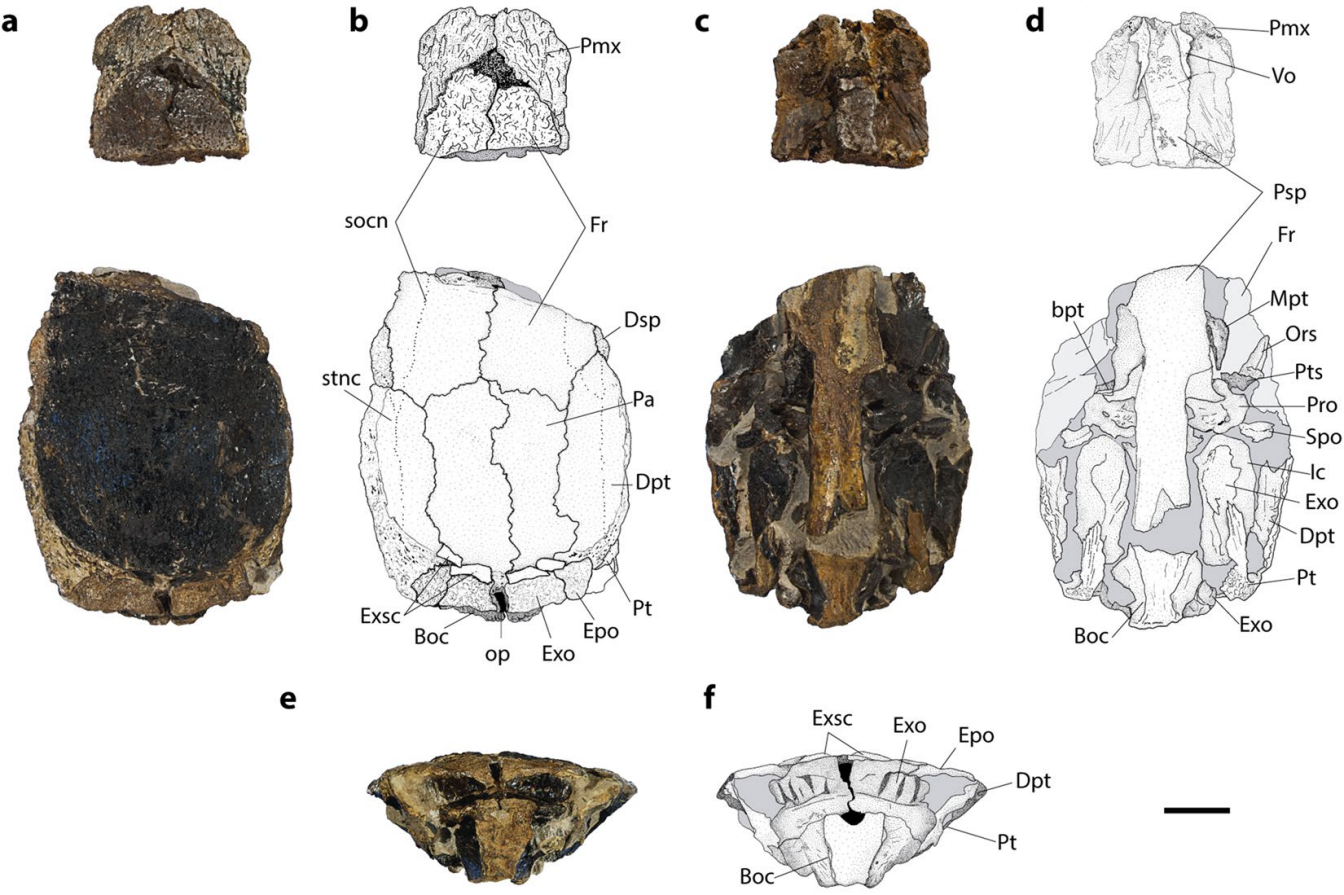
*Nhanulepisosteus mexicanus* gen. et sp. nov. Holotype, IGM 4898 in (**a**) dorsal view, (**b**) interpretative drawing, (**c)** ventral view, (**d**) interpretative drawing, (**e**) posterior view, and (**f**) interpretative drawing. Abbreviations: Boc, basioccipital; bpt, basipterygoid; Dpt, dermopterotic; Dsp, dermosphenotic; Epo, Epiotic; Exsc, extrascapular; Exo, exoccipital; Fr, frontal; Ic, intercalar; Mpt, metapterygoid; Ors, orbitosphenoid; Pa, parietal; Pmx, premaxilla; Pro, prootic; Psp, parasphenoid; Pt, posttemporal; Pts, pterosphenoid; oc, occiput; socn, supraorbital sensory canal; Spo, sphenotic; stnc, supratemporal sensory canal. Scale bars, 20 mm.

**Figure 2:**
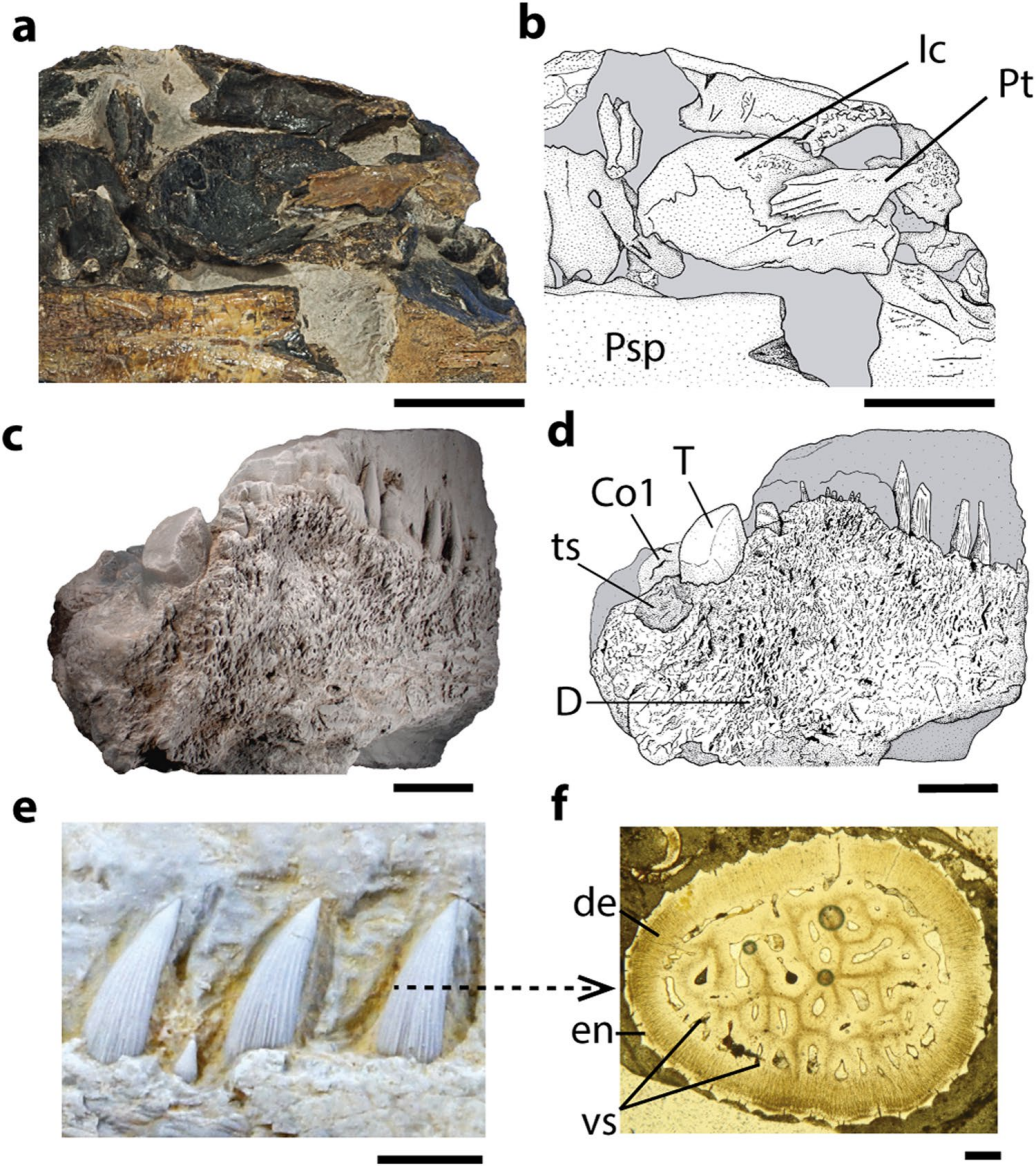
Synapomorphies of *Nhanulepisosteus mexicanus* gen. et sp. nov. (**a**) Holotype, IGM 4898, postero- ventral view of the neurocranium, (**b**) interpretative drawing, (**c**) IGM 4899, anterior view of the left dentary facing laterally, (**d**) interpretative drawing, (**e**) IGM 4901 detail of dentary teeth, **(f)** cross section in the mid part of the tooth, specimen IGM 4902, in natural transmitted light. Abbreviations: Co1, first coronoid; D, dentary; de, orthodentine; en, enamel; Ic, Intercalar; Psp, parasphenoid; Pt, posttemporal; T, large tooth; ts, tooth socket; vs, vascular spaces. Scale bars, 20 mm (**a**, **b**); 5 mm (**c**, **d**); 3 mm (**e**); 100 µm (**f**).

*Nhanulepisosteus mexicanus* gen. et sp. nov.

(Figs 1, 2, Supplementary Fig. 1)

### Etymology

Genus named after *nhanu* (old) in Mixteca language and *lepisosteus* (*lepis* (scale) + *osteus* (bone)) in Greek. The Mixtecs are an ethnic group who occupied the territory of Oaxaca, South of Mexico, including the type locality of the new species. Species is named for the country in which the fossil was collected.

### Holotype

IGM 4898, an incomplete endochondral neurocranium with dermal bones, preserved in three dimensions (Fig. 1), housed in the “Colección Nacional de Paleontología” of the Instituto de Geologia, Universidad Nacional Autónoma de México, Mexico.

### Referred material

Other specimens used in this description are: IGM 4899, left dentary; IGM 4900, part and counterpart of a partial left lower jaw presenting some dermal and pterygoid bones; IGM 4901, partial left lower jaw and lacrimomaxillary; IGM 4902, disarticulated specimen including a broken dentary, some unidentified bones and scales.

### Type locality and age

All material comes from an exposure of the Llano Yosobé deposits of the Sabinal Formation, near the town of Tlaxiaco, Oaxaca, southern Mexico. The outcrop consists of siltstones and dark bituminous shales with nodules, interbedded with limestones. A Kimmeridgian (Late Jurassic) age was inferred for the Llano Yosobé deposits based on the associated fauna, including vertebrates and invertebrates^15–17^. This lithological unit is interpreted a marine deposit, with datable ammonites.

Although the top of the Sabinal Formation has not been observed in the Llano Yosobé, the contact between the underlying Callovian - early Kimmeridgian “Caliza con Cidaris” unit and the Kimmeridgian-Tithonian Sabinal Formation is transitional at this site. The nodule bearing strata with vertebrates of the Llano Yosobé represents a sequence of about 40 m, and contains typical Kimmeridgian invertebrates including the chronostratigraphicaly important ammonites *Idoceras* and *Ataxioceras* ^18,19^, resting just above the last occurrence of the bivalves *Myophorella sologureni* (Felix, 1891) and *Gryphea mexicana* Felix, 1891, in the top of the “Caliza con Cidaris” unit.

### Diagnosis for monotypic genus and species

*Nhanulepisosteus mexicanus* differs from all other lepisosteoids by the following characters: presence of a well-developped intercalar; posttemporal penetrated by the lateral line sensory canal; posttemporal bone with a hypertrophied rod-like process on the ventral surface that connects with the intercalar; hypertrophy of the two anterior dentary teeth in relation to the immediately posterior teeth; teeth with grooves and striations of dentine distributed over almost the entire length; plicidentine folds occupying nearly the entire pulp cavity of the tooth.

### Description

The skull roof is partially preserved in the holotype (Fig. 1a, b) and comprises the premaxillae, frontals, parietals, dermosphenoids, dermopterotics, extrascapulars, and posttemporals. All bones are well ornamented on their outer surfaces, as are the dermal bones of the jaw. The ornamentation of the cranial bones is strongly developed and coarse, which greatly hinders the observation of the sensory canals.

The premaxillae are noticeably shorter than in extant gars and resemble those of the short-snouted genera *Cuneatus* and *Masillosteus* ^11^. As in other gars, the part of these bones that forms a portion of the skull roof is ornamented on its dorsal surface. The anterior portions of these bones are pierced by foramina for the olfactory nerves. The premaxillae are fixed and connect posteriorly with the frontals. The supraorbital sensory canals extend longitudinally, close to the lateral margins of the bones. The frontals are the largest bones of the skull roof, and seem to be the longest. Anteriorly, the frontals are tapering bones, presenting projections of thin bone on their lateral edges. Medially, the suture between the frontals is almost straight, with very few interdigitations. The supraorbital sensory canal connects to the supratemporal canal of the dermopterotic. Like in other lepisosteoids, the dermosphenotics are located in the postero-lateral corners of the frontals and are tightly sutured into the skull roof; they do not reach the orbital margin.

The parietals and the dermopterotics of *Nhanulepisosteus mexicanus* form a transverse series of bones tightly sutured to the other skull roof bones. Anteriorly they overlap the frontals and posteriorly they lie just in front of the extrascapulars. At least four extrascapulars are present in the holotype. The complete series should, as in other lepisosteiforms, overlie the posterior part of the dermopterotics, the epioccipital, and the exoccipitals.

The posttemporals of *Nhanulepisosteus mexicanus* are complex bones that underlie the posterior margin of the extrascapsular series. The bones bear a hypertrophied rod-like process on the ventral surface, running anteriorly and connecting with the intercalar bone of the braincase. In contrast to known lepisosteids, the posttemporal appears to be penetrated by the lateral line sensory canal.

The braincase presents the typical lepisosteid pattern: loss of opisthotic and basisphenoid, no supraoccipital, and no posterior myodome. The orbitosphenoid is median, Y-shaped. The two wings are fused rostrally on the midline of the skull forming a tunnel for accommodating the olfactory nerves. Posteriorly, each wing of the bone suture to the pterosphenoids.

The prootic extends on the lateral anterior wall of the braincase and is pierced by a large foramen for the hyomandibular trunk of the facial nerve. Laterally the prootic articulates with the sphenotic. Anteriorly, a well-developed basipterygoid process is present. It is formed by the prootic and by the ascending wing of the parasphenoid, and shows an antero-lateraly directed articular facet. The sphenotic is sutured to the underside of the dermopterotic.

The exoccipitals extend from their anterior part in where they border the vagus nerve foramen, posteriorly to the rear of the neurocranium, where they suround the foramen magnum. Antero-dorsally, the exoccipitals suture with the well-developed intercalar bones. Posteriorly, although partially hidden by the ventral process of the posttemporals, the exoccipitals articulate with the epioccipitals. The intercalars are similar to those of halecomorphs and teleosts. They are well developed bones that extends anteriorly to the prototics and are slightly sutured ventrally to the exoccipitals.

The epiotics are sutured to the dorsal margin of the exoccipitals and form the postero-dorsal part of the braincase. In posterior view, they nearly meet their antimere on the midline. In lateral view they extend dorsally to meet the dermopterotic. The basioccipital is a short bone that extends below the exoccipital and seems to incorporate two vertebrae.

Paired vomers are partially preserved on the holotype (Fig. 1c, d). They contact each other medially and are firmly sutured to the parasphenoid. The parasphenoid is partially preserved in the holotype. Like other lepisosteoids, there is a lateral wing forming, together with the anterior edge of the prootic, the basipterygoid process. This process articulates with the metapterygoid. Posterior to this process there is a notch for the posterior palatine branch of the facial. The posterior end of the parasphenoid is forked.

A fragment of the upper jaw is preserved. An ornamented bone, probably a premaxilla, bordered by a small, rectangular lacrimomaxillary bone, with its outer row of very small teeth can be seen (Supplementary Fig. 1c, d). Presence of lacrimomaxillary bones is a derived character of lepisosteid gars.

The dentary is an elongate bone, medially recurved on its anterior end. The bone increases its height in the rear (Supplementary Fig. 1d–h). Its outer surface is ornamented with longitudinal striations, and bears the pores of the mandibular sensory canal. The dentary bears two rows of teeth: a marginal one presenting numerous marginal teeth and a mesial one with large and robust fangs (Fig. 2c–e). The two most anterior teeth of the dentary are hypertrophied being at least five times larger than those teeth lying immediately posterior (Fig. 2c, d).

The anterior coronoid is a medially curved and anteriorly broad bone that sutures with its antimerous at the symphysis (Supplementary Fig. 1a, b). It bears small teeth, similar in size to those of the marginal teeth row of the dentary. Very few bones of the palatal complex are preserved. Among them, an anterior dermopalatine, with its strongly developed teeth, the posterior part of the ectopterygoid, and a metapterygoid with a large articular surface for the basipterygoid process (Supplementary Fig. 1d, e).

Contrary to other gars with plicidentine, the larger teeth of *Nhanulepisosteus* have ridges distributed over almost their entire length (Fig. 2e). As observed in cross sections, these ridges are due to more or less thickening of the enamel layer giving thus a crenelated aspect. Under the enamel layer there is a relatively thick layer of dentine constituted of vascular canals surrounded by dentinal tissue and crossed by orthogonal odontoblastic canalicles. Dentine folds occupy nearly the entire pulp cavity of the tooth, and thus represent plicidentine. This dentine is crossed by parallel odontoblastic canalicles (=orthodentine) starting from the wall surface of vascular canals. This folded dentine, filling almost the entirety pulp cavity, closely resembles the dendrodont dentine^20^. The enameloid does not penetrates inside the dentine plies (Fig. 2f), sharply contrasting with the pattern found in modern gars^11,21^. The smaller teeth also have an external ridged enamel layer that overlays the dentine layer. However, on these teeth, the pulp cavity is totally empty and without plies.

The few scales associated with our material are of the ganoid lepisosteoid-type. Histologically the scales show two superposed layers: an external hypermineralised layer of unstratified ganoin that is less than 100 µm thick and a basal plate of cellular bone crossed by numerous canalicules of Williamson, and that appears practically avascular (Supplementary Fig. 1i, j). Contrary to the condition found in obaichthyids, no dentine was observed between the ganoin layer and the basal plate.

### Phylogenetic analysis

**Figure 3:**
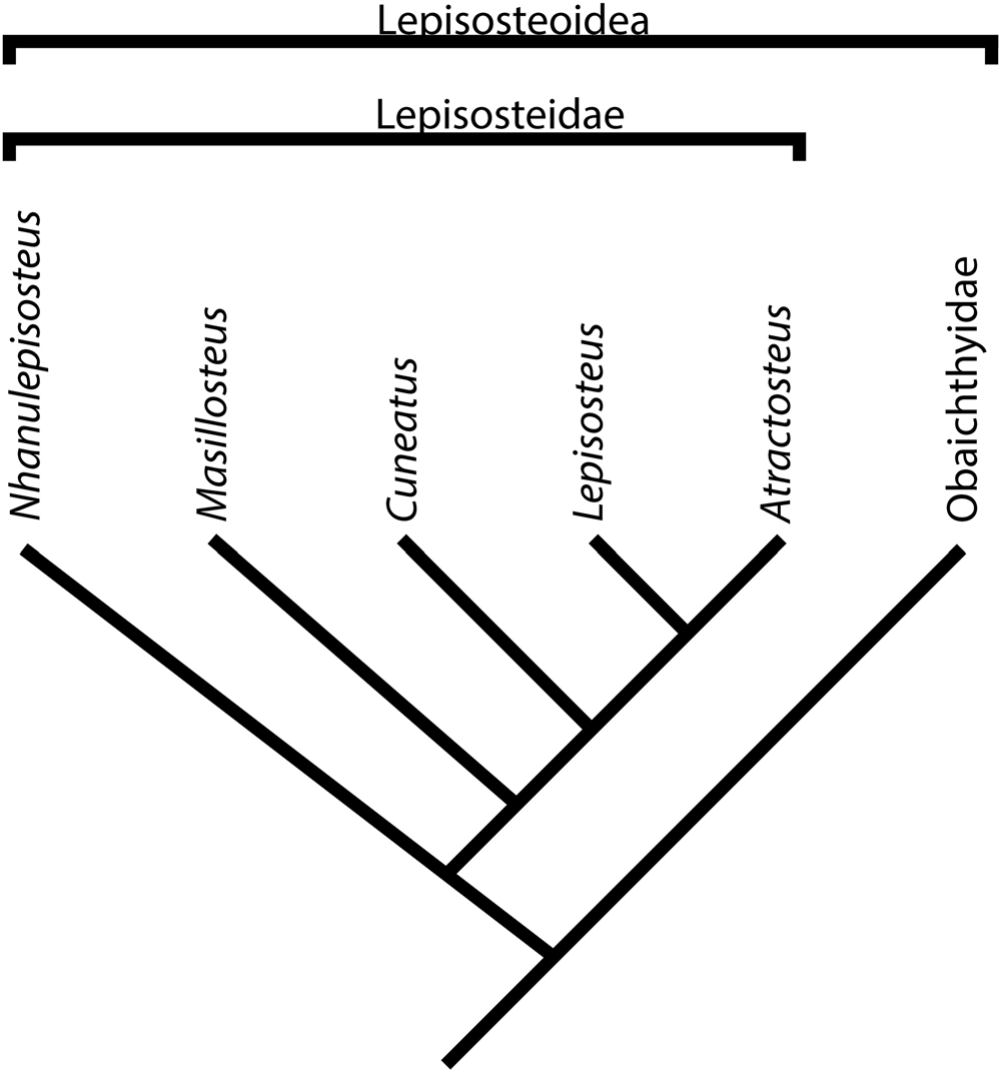
Simplified Phylogeny of the Lepisosteoidea. Simplified hypothesis based on the strict consensus tree inferred from 176 most parsimonious trees (consistency index = 0.683; retention index = 0.903) modified from Grande^11^ (see Supplementary Figs 2 and 3).

A phylogenetic analysis was performed in PAUP* 4.0b10^22^ and TNT software package^23^ using a data set based on Grande^11^. The inclusion of *Nhanulepisosteus* did not change the interrelationships among the major clades of lepisosteoids^11^ obtained in that previous study. We obtained 176 most parsimonious trees. The strict consensus tree hypothesizes the presence of two well resolved clades of gars among the Lepisosteoidea: Lepisosteidae and Obaichthyidae. *Nhanulepisosteus* falls as the sister taxon of the rest of the Lepisosteidae (Fig. 3 and Supplementary Fig. 2, 3).

## Discussion

*Nhanulepisosteus* is unambiguously a member of Lepisosteoidea, and presents a combination of both typical modern lepisosteid features and plesiomorphic characters. Synapomorphies of Lepisosteidae^11^ observed on *Nhanulepisosteus* are: 1- the great number of extrascapulars, 2- the supraorbital canal incorporated in the premaxilla, 3- presence of lacrimomaxillaries, 4- the junction of supraorbital sensory canal with infraorbital canal within the dermopterotic, 5- a laterally sliding articulation between metapterygoid and the basipterygoid process, 6- presence of plicidentine on teeth, and 7- Lepidosteoid-type scales. However, two conspicuous characters, 1- the presence of a well-developed intercalar bone and 2- a posttemporal with a hypertrophied rod-like process on its ventral surface, bearing the sensory canal are not found in any known lepisosteids. Instead, two characters are shared, in part, with obaichthyids and strongly resemble those of halecomorphs and basal teleosts, and presumably represent retained plesiomorphies.

The absence of an intercalar is generally treated, in current literature, as a synapomorphy of Ginglymodi^9,24,25^. Although unknown in the majority of ginglymods, including the extant gars, an individualized intercalar bone had been described in the Lepisosteoidei *Thaiichthys buddhabutrensis* from the ? Late Jurassic- Early Cretaceous of Thailand^10^. In *Dentilelepisosteus*, from the Early Cretaceous of northeastern Brazil and Late Cretaceous of Morocco, there is a somewhat prominent element, the “intercalary process”, fused to the exoccipital that was considered homologous with the intercalar^10^. The presence of an individualized intercalar in *Nhanulepisosteus* confirms the existence of this bone in the more primitive gars. The presence of this bone becomes therefore a synapomorphy of neopterygians, as proposed by Grande^11^, the intercalar bone being lost several times within Ginglymodi.

The posttemporal of extant gars contains neither the lateral line canal nor a ventral process that connects to the intercalar. In *Nhanulepisosteus*, the posttemporal is crossed by the lateral line canal and there is an important ventral rod-like process linking the intercalar, similar to that found in halecomorphs^26^. A posttemporal penetrated by the lateral line and with a long rod-like process is also found on the obaichthyid *Dentilelepisosteus* ^11,14,27^, halecomorphs and teleosts^11,26,28^.

Apart from these retained primitive features, *Nhanulepisosteus* presents some of the derived skull patterns found in all lepisosteoids such as the absence of a posttemporal fossa, a basisphenoid, and of a posterior myodome. *Nhanulepisosteus* also presents the derived features found in lepisosteids such as fixed lacrimomaxillary bones, a dentary with different rows of long sharp teeth and teeth with plicidentine *contra* the obaichthyid pattern in which there is a free maxillary, with a unique row of minute teeth on its upper and lower jaws, and no plicidentine on their teeth.

The features observed on the jaws of *Nhanulepisosteus* suggest that this taxon had the specialized predatory feeding mechanism of the extant gars^11,29^. This specialization of the jaws added to the adaptations of the braincase, allow us to hypothesize that the body plan of gars, at least in terms of skull geometry, already existed in the Late Jurassic, thus confirming the morphological conservatism of this group (Supplementary Fig. 4).

**Figure 4:**
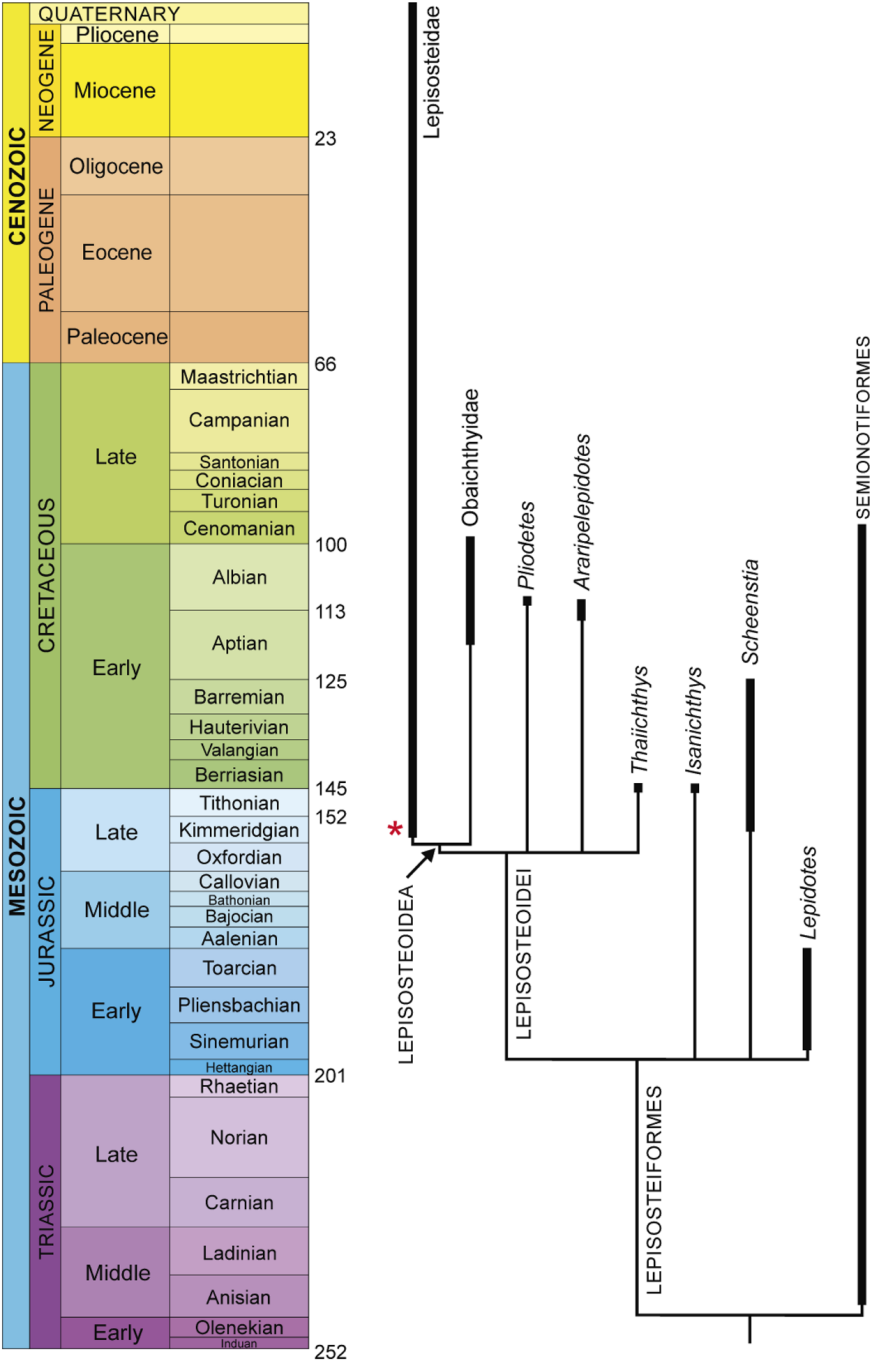
Simplified geological time range of ginglymodians. Distribution through geological time modified from López-Arbarello^9^. Asterisk indicates the age of *Nhanulepisosteus mexicanus* gen. et sp. nov. Thick lines represent sampled fossil record, while thin lines highlight the ghost lineages.

Previously, the most ancient records of Lepisosteoidei were from Early Cretaceous beds of Western Gondwana and Thailand^9,10^. When we calibrate *Nhanulepisosteus* against the geological age of fossil taxa, a basal lepisosteoid radiation is revealed by the presence of four ghost lineages (*Araripelepidotes*, *Pliodectes*, and *Thaiichthys* and Lepisosteoidea; Fig. 4) that must have originated by at least the Kimmeridgian. All these forms appear later in the fossil record, in Early Cretaceous beds referred to the Aptian/Albian (125–100 Ma). Therefore, *Nhanulepisosteus* extends the evolutionary origins of these lineages at least 46 Myr before their first appearance in the fossil record. It also extends the fossil record of lepisosteids by approximately 57 million years. The confirmation of a lepisosteoid radiation in the Late Jurassic contrasts with previous interpretations regarding the early evolution of gars^9,11^. The presence of *Nhanulepisosteus*, a basal lepisosteid gar, in the Kimmeridgian nests obaichthyid gars at least in the Late Jurassic pointing to a possible diversification of this group prior to Kimmeridgian.

Finally, *Nhanulepisosteus* also sheds light on the evolution of the ecology of gars. This new taxon appears to have inhabited a fully marine environment with mixed components from the Tethys and the Pacific Ocean^15–17^. Apart from rare occurrences in brackish and even marine waters of the species *Atractosteus spatula*, extant lepisosteids primarily inhabit freshwater environments^11^. The same appears to be true for fossil lepisosteids and obaichthyids. *Nhanulepisosteus* is considered a marine taxon, but all other fossil lepisosteids are freshwater. Therefore, there is an implication that the invasion into freshwater most likely occurred between the Late Jurassic and Early Cretaceous.

## Methods

### Phylogenetic analysis

Present phylogenetic analysis is based on Grande^11^ data set, which constitutes the most comprehensive analysis regarding the phylogeny of Lepisosteoidea. The matrix was only modified by the inclusion of *Nhanulepisosteus* (Supplementary Note 1). The phylogenetic analysis was performed using the parsimony method in PAUP* 4.0b10^22^ and performed the analysis with ACCTRAN optimization. All characters were equally weighted and treated as unordered. This produced 176 most parsimonious trees (MPTs) of 183 steps (consistency index = 0. 683, retention index = 0. 903). Our resulting strict consensus tree displays a total of 23 components (Supplementary Figs 2, 3) and agrees with previous analyses, including support for the neopterygians and a monophyletic holostean clade. Among the holosteans, the ginglymodians are confirmed as monophyletic. The macrosemiids (*Macrosemius*) diverge earliest, followed by *Semionotus* being the sister group of a monophyletic Lepisosteoidea, in which the early Cretaceous Obaichththyidae is the sister taxon of the Lepisosteidae^11^. Bootstrap values were calculated in PAUP. Bremer Decay values were calculated in TNT software package^23^ (Supplementary Fig. 2).

### Histological preparation

Ground cross sections were made using the methodology of Matrajt *et al*.^30^, modified by Meunier *et al*.^31^.

### Nomenclatural act

This published work and the nomenclatural act it contains have been registered in ZooBank, the proposed online registration system for the International Code of Zoological Nomenclature (ICZN). The ZooBank LSID (Life Science Identifier) can be resolved and the associated information viewed through any standard web browser by appending the LSID to the prefix ‘http://zoobank.org/’. The LSID for this publication is: urn:lsid:zoobank.org:pub:290F8440-470F-4AD4-96C6-5C3947A40C40.

### Data availability

The authors declare that all data supporting the finding of this research are available within the paper and its Supplementary Information.

## Electronic supplementary material

Supplementary information
