# Supplementary material for: Earliest known lepisosteoid extends the range of anatomically modern gars to the Late Jurassic
Source: Sci Rep. 2017 Dec 19;7:17830. doi: 10.1038/s41598-017-17984-w (PMC5736718; doi:10.1038/s41598-017-17984-w)
Supplement: Supplementary file 1 — Supplementary information [file 41598_2017_17984_MOESM1_ESM.pdf]

Earliest known lepisosteoid extends the range of anatomically modern gars to the Late Jurassic

Paulo M. Brito[1*], Jésus Alvarado-Ortega[2] and François J. Meunier[3]

1 Departamento de Zoologia, Instituto de Biologia, Universidade do Estado do Rio de Janeiro, Rio de Janeiro, Brazil

2 Paleontología, Instituto de Geología, Universidad Nacional Autónoma de México, Ciudad Universitaria, Coyoacán, Distrito Federal, 04510 Mexico.

3 UMR 7208 (CNRS-IRD-MNHN-UPMC), BOREA, Département des Milieux et Peuplements aquatiques, Muséum National d'Histoire Naturelle, CP26, 43 rue Cuvier, 75231 Paris cedex 05, France.

*Correspondence should be addressed to P.M.B. (pbritopaleo@gmail.com)

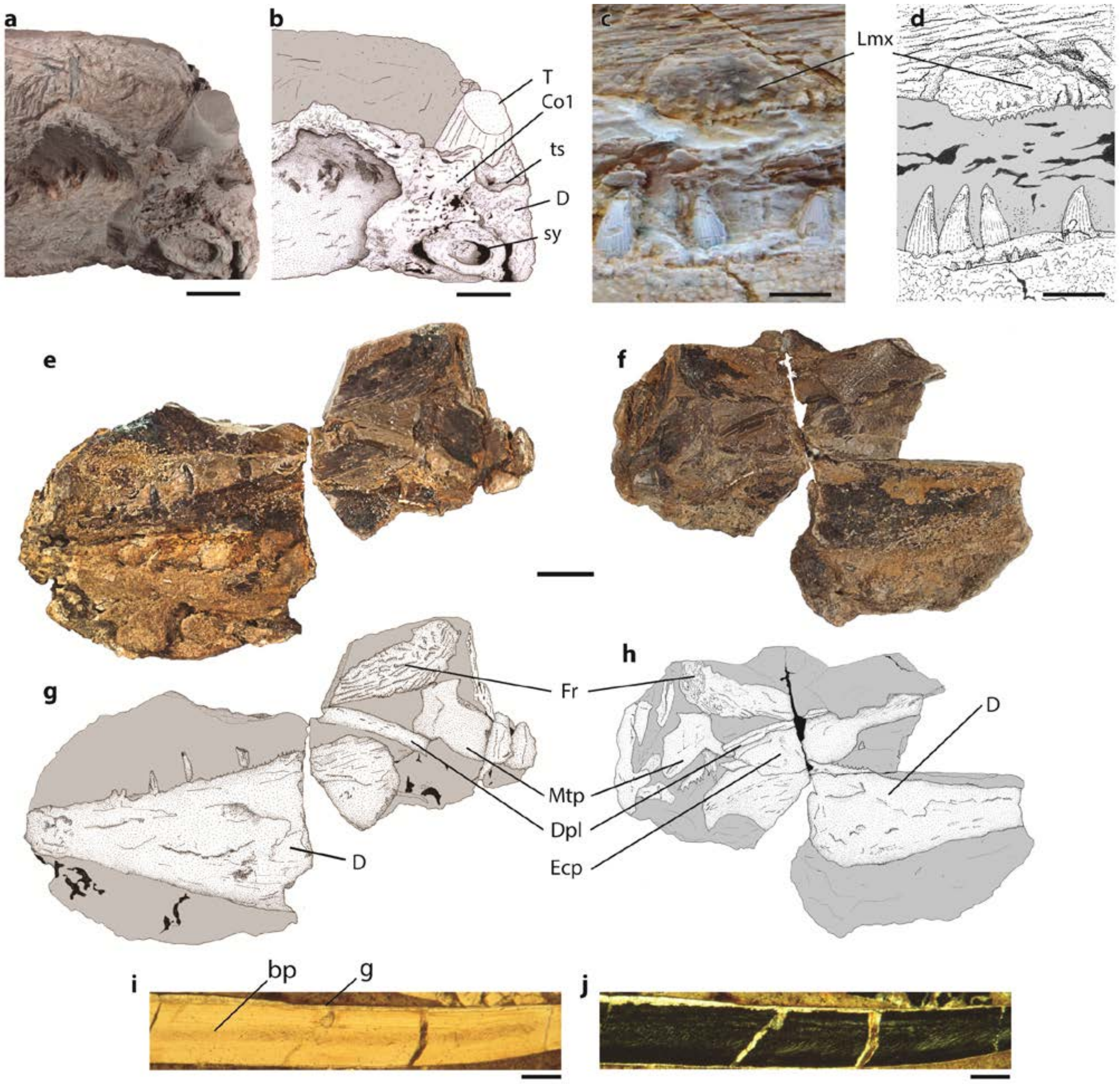

Supplementary Figure 1 | *Nhanulepisosteus mexicanus* gen. et sp. nov. (**a**) IGM 4899, anterior view of the left dentary facing medially and (**b**) interpretative drawing, (**c**) IGM4901, partial left jaw, showing the lacrimomaxillary, **(d)** interpretative drawing, (**e**) IGM 4900, main slab with some dermal and pterygoid bones and (**f**) counterpart of the same slab, (**g**) interpretative drawing of "e", (**h**) interpretative drawing of "f", (**i**) IGM 4902, scale histology in natural and (**j**) polarized transmitted light. Abbreviations: bp, basal plate; Co1, first coronoid; D, dentary; Dpl, dermopalatine; Ecp, ectopterygoid; Fr, frontal; g, ganoin; Lmx, lacrymomaxilla; Mtp, metapterygoid; sy, symphysis; T, large tooth; ts, tooth socket. Scale bars, 5 mm (**a, b**); 3 mm (**c, d**); 20 mm (**e- h**); 100 µm (**i**, **j**).

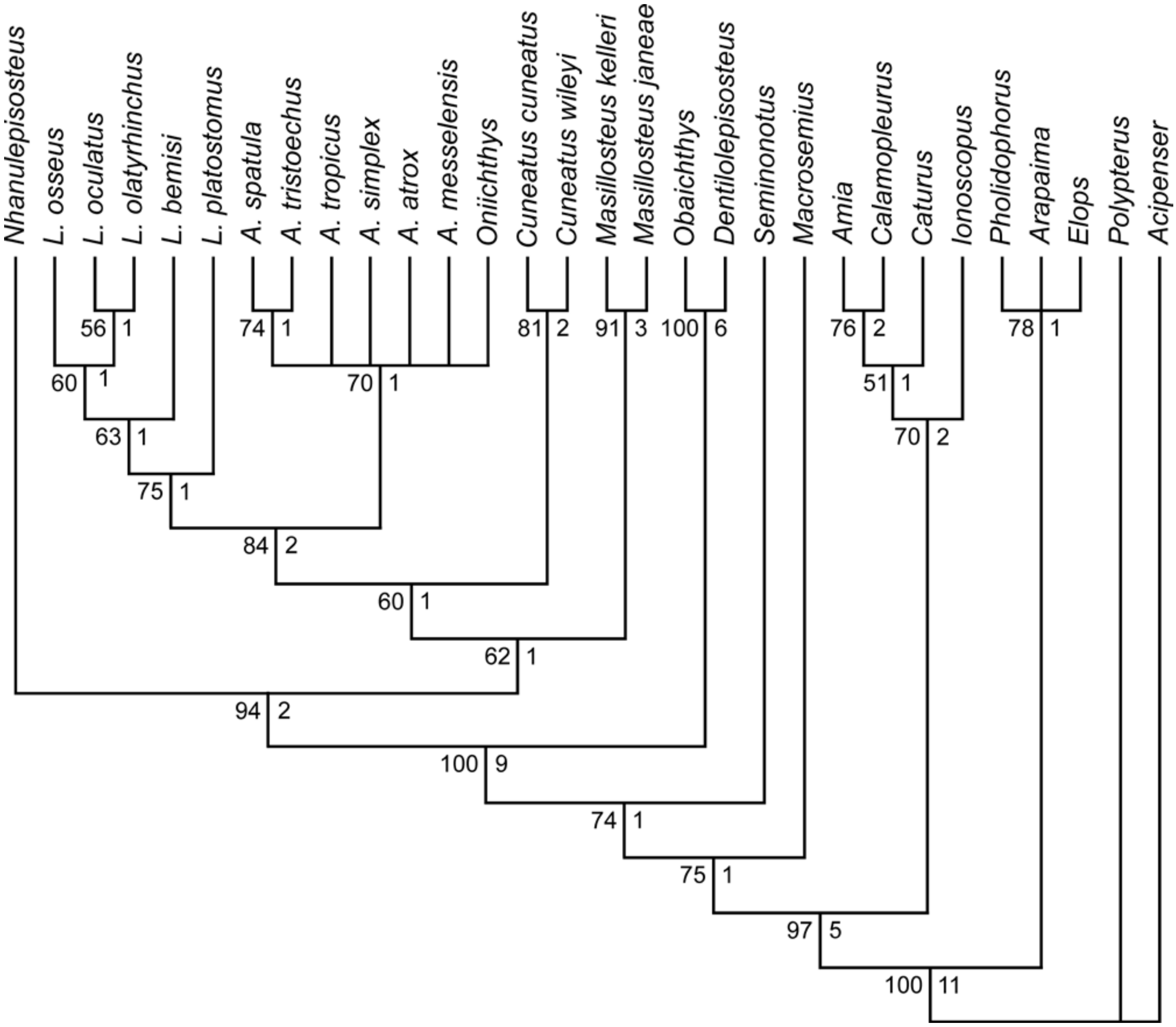

Supplementary Figure 2 | Strict consensus tree of the 176 shortest trees (183 steps) for 30 taxa and 105 equally weighted characters showing the phylogenetic position of *Nhanulepisosteus mexicanus* gen. et sp. nov., modified from Grande[11]. Digits on the right side of the nodes indicate Bremer decay; digits on the left side of the nodes indicate percentage bootstrap support. We considered on the present analysis *Atractosteus falipoui* (Cavin & Brito, 2001) as *Oniichthys.*

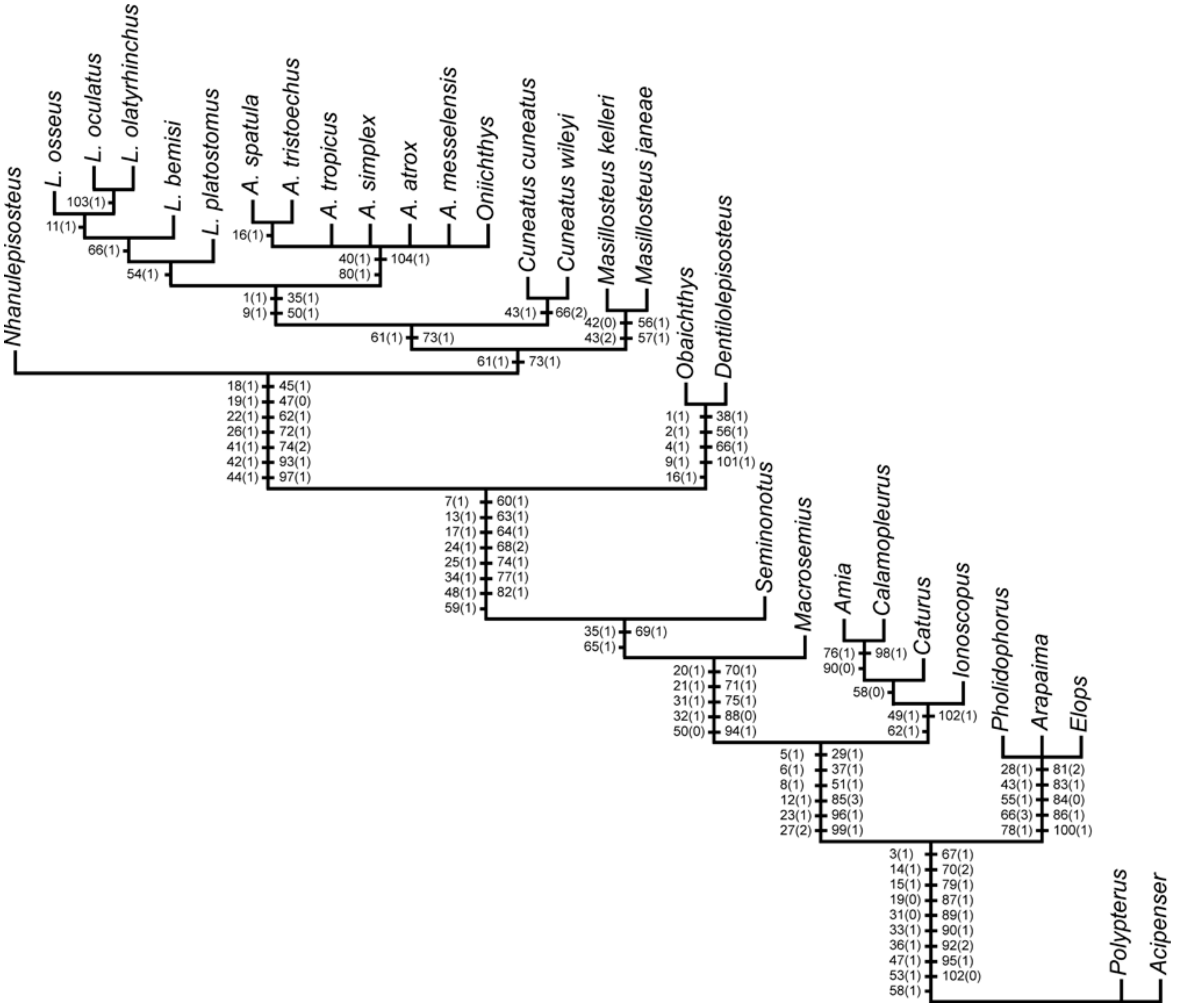

Supplementary Figure 3 | Strict consensus tree of the 176 shortest trees (183 steps) for 30 taxa and 105 equally weighted characters showing the apomorphic list giving character changes for nodes. Characters are based on Grande[11].

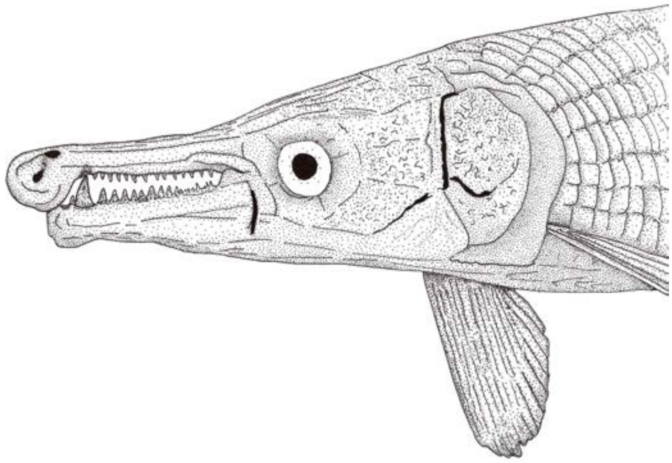

Supplementary Figure 4 | Cranial reconstruction of the living lepisosteid *Nhanulepisosteus mexicanus* gen. et sp. nov.

Supplementary Note 1. Scoring for *Nhanulepisosteus mexicanus* in the data matrix of Grande [11]

Nhanulepisosteus mexicanus gen. and sp. nov.

?0??111100 ??1??0???? ?1111??010 ?11??11?1? 11???????? ???0000?11 ?????????? ????1????? ?1???????? 02???????? ?0??0
